# Anterior Cruciate Ligament Reconstruction Improves Functional Scores and Quality of Life in Patients Older Than 50 Years of Age

**DOI:** 10.1016/j.asmr.2023.100806

**Published:** 2023-10-19

**Authors:** Batuhan Çokyaşar, Ozan Altun, Uygar Daşar

**Affiliations:** a Department of Orthopedics and Traumatology, Merzifon Kara Mustafa Paşa State Hospital, Amasya, Turkey; b Department of Orthopedics and Traumatology, Çankırı State Hospital, Çankırı, Turkey; c Department of Orthopedics and Traumatology, Karabuk University, Karabuk, Turkey

## Abstract

**Purpose:**

To evaluate quality of life changes and functional outcomes of anterior cruciate ligament reconstruction in patients older than 50 years of age with anterior cruciate ligament injury.

**Methods:**

Patients who were older than 50 years of age and had undergone anterior cruciate ligament reconstruction with single-bundle hamstring tendon auto graft surgery between January 2016 and February 2018 were identified. Preoperative Tegner activity scores, Lysholm knee scores, International Knee Documentation Committee (IKDC) 2000 scores, and Short-Form 36 scores were compared with results that were documented 1 year after surgery.

**Results:**

A total of 35 patients were included (20 male/15 female; mean age 52 years [range 50-59 years]). Patients’ preoperative mean Tegner score was 1.48. Mean Tegner score at 1-year follow-up was 3.82. Preoperative mean Lysholm score was 45.8. Postoperative mean Lysholm score was 88. Preoperative mean IKDC 2000 score was 33. Postoperative mean IKDC 2000 score was 82. All of these changes were statistically significant (*P* < .05). All of the parameter changes at Short Form-36 except for role limitations due to emotional problems were statistically significant.

**Conclusions:**

Improved functional knee scores, quality of life, and psychological status were achieved at anterior cruciate ligament reconstruction in patients older than 50 years of age.

**Level of Evidence:**

Level IV, therapeutic case series.

Anterior cruciate ligament (ACL) rupture is one of the most common injuries in middle-aged athletes and can cause tibiofemoral instability, resulting in patients being unable to return to preinjury activity levels. ACL reconstruction is one of the most commonly performed procedures in orthopaedic practice, with a reported success rate of 85% to 95% in young patients.^1^

Historically, conservative treatment options have been preferred in middle-aged patients with ACL injury, and they have been asked to change their physical activities.^2^^,^^3^ However, recent studies have reported that the results of conservative treatment are not good and there is an increased risk of residual instability.^4^^,^^5^ It has been observed that increased residual instability with conservative treatment increases cartilage lesions and the risk of meniscal injury.^5^, ^6^, ^7^ It is well known that especially active patients in all age groups are increasingly reluctant to limit their sports activities and demand full restoration of all knee functions.^8^, ^9^, ^10^

In contrast, there is concern that middle-aged patients treated with surgery may have worse outcomes, with a greater complication rate and risk of progression of chondral degeneration.^8^^,^^11^, ^12^, ^13^ Other concerns include the effects of increasing age on bone quality, graft integration, and healing potential.^14^^,^^15^ Uncertainties and controversies persist regarding the choice of treatment for ACL injuries in this population due to insufficient clinical evidence.^16^

The purpose of our study is to evaluate quality of life changes and functional outcomes of ACL reconstruction in patients older than 50 years of age with ACL injury. We hypothesized that arthroscopic ACL reconstruction would a successful treatment option and would provide functional recovery in patients with ACL injury older than 50 years of age.

## Methods

Patients older than the age of 50 years who underwent arthroscopic reconstruction surgery using hamstring tendon autograft for ACL tear between January 2016 and February 2018 were analyzed retrospectively. Patients with Kellgren–Lawrence stage 4 osteoarthritis, patients with ACL re-rupture, patients treated with grafts other than hamstring tendon autografts, patients with a follow-up period of less than 1 year, patients with a history of previous same-sided knee surgery, and patients younger than 50 years of age were excluded. Patients who were older than 50 years of age who had ACL injury that was treated with single-bundle hamstring tendon autograft were included in the study. Patients without concomitant ligament injury and patients without varus–valgus deformity were included in the study. Patients diagnosed with meniscopathy that required intervention during arthroscopy (eg, partial menisectomy, meniscus repair) and chondral pathology that required intervention (eg, microfracture, mosaicplasty) were excluded from study. Informed consent form was obtained from all patients included in the study.

ACL tear diagnosis was made with combination of 3 factors: physical examination (positive for Lachmann test and/or anterior drawer test), patient complaints (instability, discomfort), and magnetic resonance imaging findings (complete tear on magnetic resonance imaging).

Following diagnosis, patients were given 3 months of conservative treatment that included nonsteroidal anti-inflammatory drugs and physical therapy. Patients who were unsatisfied with results of conservative treatment were enrolled for surgical intervention and included in this study.

Age, sex, height, weight, trauma leading to injury, time of injury, history of comorbidities, and regular medication history were recorded preoperatively. Preinjury, postinjury, and postoperative Tegner activity index of the patients were noted. The Lysholm knee score, International Knee Documentation Committee (IKDC) 2000 and Short Form-36 (SF-36) forms were filled out preoperatively and 1-year postoperatively.

Cefazolin sodium was administered intravenously 1 hour before anesthesia for prophylactic antibiotherapy. Postoperative antibiotic prophylaxis was continued for 24 hours. All patients received enoxaparin sodium for thromboembolic prophylaxis. All patients were operated by a single surgeon to optimize outcomes. A quadrupled hamstring tendon autograft was used in all patients. The tendon graft was placed in the femoral tunnel drilled from an anteromedial arthroscopy portal with an ENDOBUTTON (Smith & Nephew, Andover, MA) and fixed at the tibial tunnel using a bioabsorbable screw. All patients were allowed as much load as they could tolerate postoperatively and were enrolled in a closed-chain exercise program. Running was allowed at 4 months and return to sportive activities was allowed at 6 months’ postoperatively.

### Statistical Analysis

Statistical evaluation of the data was performed using Statistical Package for the Social Sciences (SPSS) for Windows, version 20.0 (IBM Corp., Armonk, NY). For numerical variables, data are presented as mean ± standard deviation and minimum-maximum values. The Shapiro–Wilk normality test was used to determine whether our data fit the normal distribution. Two-sample *t*-test was performed for the data fitting the normal distribution, and Mann–Whitney *U* test was performed for the data not fitting the normal distribution. Tests were considered significant if the *P* value was less than .05.

## Results

Of the 35 patients included in the study, 20 were female and 15 were male. The mean age of the patients was 52.4 (50-59) years. In total, 16 patients were operated on the left side and 19 on the right side. ACL rupture occurred in 28 patients after a simple fall/sprain and in 7 patients as a result of rotational trauma during sportive activity.

Mean height of patients was 169.3 cm, and mean weight was 78.6 kg. Six patients had hypertension, 3 patients had diabetes mellitus, 1 patient had allergic asthma, and all of these patients’ diseases were under control with medication.

Preinjury mean Tegner activity scale score was 4.1 (range 2-7), preoperative mean score was 1.4 (range 0-4), and postoperative mean score was 3.8 (range 2-7). These changes were statistically significant (*P* = .000). Patients who were not involved in sportive activities have successfully returned to their preinjury daily activity levels. Three of 7 patients who played recreational sports have discontinued their sporting activities due to fear of reinjury. The remaining 4 patients returned their sporting activities with no problems. Preoperative and 12 months’ postoperative functional knee scores can be seen in Table 1.

**Table 1:** Preoperative and Postoperative Functional Knee Scores

Parameter	Preoperative Mean Value (Standard Deviation)	12 Months’ Postoperative Mean Value (Standard Deviation)	*P* Value
Tegner activity score	1.48 (1.42)	3.82 (1.56)	.000
Lysholm knee score	45.83 (11.3)	88.3 (6.5)	.000
IKDC 2000	33.5 (5.3)	82.0 (9.8)	.000

IKDC, International Knee Documentation Committee.

Preoperative mean Lysholm score was 45.8 (31-67). Postoperative mean Lysholm score was 88 (75-100). This change was statistically significant (*P* = .000).

Preoperative mean IKDC 2000 score was 33.5 (27.6-41.7). Postoperative mean IKDC 2000 score was 82 (71-100). This change was statistically significant (*P* = .000).

The subgroups of the SF-36 questionnaire are shown in Table 2. The quality of life of the patients was statistically significantly greater in the first postoperative year (*P* = .000).

**Table 2:** Preoperative and Postoperative Short Form-36 (SF-36) Values

Parameter (SF-36)	Preoperative Mean Value (Standard Deviation)	12 Months ‘Postoperative Mean Value (Standard Deviation)	*P* Value
General health	75.7 (5.3)	92.4 (7.2)	.000
Mental health	74.8 (13.8)	86.1 (10.8)	.000
Change in general health	30.0 (18.9)	89.2 (12.5)	.000
Pain level	58.9 (12.0)	88.0 (7.7)	.000
Energy level	67.7 (11.5)	89.0 (8.8)	.000
Social functioning	79.6 (15.1)	92.5 (10.5)	.000
Physical functioning	56.1 (6.7)	87.7 (6.5)	.000
Role limitations due to emotional problems	76.4 (27.8)	86.6 (21.7)	.230
Role limitations due to physical health	39.2 (24.4)	87.1 (16.4)	.000

In the early postoperative period, 1 patient had superficial wound infection on the donor site and was treated with oral antibiotherapy. Postoperatively, 3 patients complained of anterior knee pain, which resolved completely at the 1-year follow-up. No thromboembolic complications developed in any of our patients. None of the patients had instability or findings compatible with re-rupture at the first-year postoperative outpatient clinic visits.

## Discussion

The most important finding of this study is that we observed a significant increase in knee functional scores (Lysholm, IKDC, Tegner activity score) after arthroscopic ACL reconstruction in patients older than 50 years of age with ACL tears. We advocate that ACL reconstruction surgery should be performed in patients older than 50 years of age, especially if they have an active lifestyle. In patients older than 40 years of age, the results of ACL reconstruction have been investigated many times and have been adopted as routine treatment.^8^^,^^17^, ^18^, ^19^, ^20^, ^21^ However, there are few studies showing the results of ACL reconstruction in patients older than 50 years. Previous studies have reported similar results to ours. First, Blyth et al.^22^ reported that ACL reconstruction was a successful and safe treatment option in their study of 30 patients older than 50 years of age. Later studies reported similar results and recommended surgical treatment of knee instability due to ACL insufficiency in patients older than 50 years.^16^^,^^23^^,^^24^ We estimate that our results are comparable with younger age groups. To our knowledge, there is only one study in the literature comparing outcomes in young and middle-aged patient groups, and ACL reconstruction in patients older than 50 years has been reported to be a safe surgical procedure with good to excellent results.^25^ In this context, there is a need for randomized controlled trials comparing the outcomes of ACL reconstruction surgery between middle-aged and younger patient groups. In 1983, Noyes et al.^26^ reported that one third of patients diagnosed with ACL rupture lived without making any changes in their lives, one third compromised their quality of life by decreasing their activity levels, and one third refused to compromise and preferred surgical reconstruction. However, over the years, the expectations of patients have increased with modern life. Seng et al.^8^ reported that middle-aged patients prefer to have a stable knee rather than living with an unstable knee, considering the complications that may develop. In a study published from Sweden, it was shown that middle-aged patients preferred to undergo surgery in order to continue their daily life activities regularly instead of living with decreased knee function and instability.^27^ In our study, the average Tegner activity score was 4.2 before surgery, 1.8 after injury, and 3.8 in the first postoperative year, which were statistically significant and showed that the patients reached the preinjury activity level. This is an indication for ACL reconstruction in itself and shows that when deciding to perform ACL reconstruction in middle-aged patients, we should consider not only patients who are involved in sportive activities but also patients whose activities of daily living are restricted.

Although the rate of return to sports after ACL reconstruction is not clearly known, it varies between 30% and 100% in the literature.^28^, ^29^, ^30^, ^31^ Ovigue et al.^31^ reported that 86% of patients older than 50 years of age returned to sports after surgery and 51% returned to preinjury sports levels. Panisset et al.^32^ reported that 83% of patients returned to sports and 50% returned to preinjury sports levels. In our study, 3 patients (8.57%) terminated their sportive activities due to fear of reinjury and the rate of return to sportive activities was observed to be compatible with the literature.

We observed that ACL reconstruction positively affects patients’ psychosocial states as well as their knee functions. In our study, Tegner activity score decreased significantly after injury compared with preinjury levels and daily activity levels decreased significantly. As a result, patients reported that they switched to a sedentary lifestyle, gained weight, and avoided exercising again. As shown in Table 2, both general health and mental health parameters of the SF-36 form improved significantly after ACL reconstruction. In patients older than 50 years of age, ACL reconstruction should be considered not only to improve knee function but also to increase the energy level, mental health, and social functioning of the patients. We concluded that the quality of life of patients whose ACL insufficiency was resolved was significantly improved. In the light of this information, we think that surgical treatment should be preferred in patients older than age of 50 years for both a stable knee and a greater quality of life.

In the past, ACL reconstruction has been avoided in middle-aged patients because of the fear for high complication rates. Conservative treatment was advocated with the thought that complications such as postoperative arthrofibrosis, limitation in knee range of motion, infection, poor wound healing, poor graft integration, and thromboembolic events would be seen more frequently in middle-aged patients than in younger patients.^21^ However, studies have refuted this view.^20^^,^^33^^,^^34^ Salesky et al.^35^ compared the complications of ACL reconstruction in patients older than younger than 50 years of age and showed that medical complications were more common in the middle-aged patient group, whereas surgical complications were more common in the younger patient group. In contrast, other studies have reported similar complication rates in young and middle-aged patients.^36^, ^37^, ^38^ In contrast to these studies, Maletis et al.^39^ reported greater revision rates in patients younger than 21 years compared with patients older than 40 years. They attributed this result to the high postoperative expectations and activity levels of young patients. We did not encounter any re-rupture in any patient in our study. We attributed the reason why we did not encounter any re-rupture cases to the relatively low activity level of the middle-aged patient group compared with younger patients and the short follow-up period.

Salesky et al.^35^ reported that thromboembolic complications may be observed more in the middle-aged group compared with the younger age group and recommended thromboembolic prophylaxis. In the same study, infection rates were similar in the young and middle-aged patient groups. In our study, no thromboembolic event developed in any patient. We explain this with the administration of enoxaparin sodium to all patients. In addition, infection in the donor site was seen in only 1 patient (2.85%) in our study, and we think that middle age group is not a risk factor for the development of infection after ACL reconstruction.

Recent studies in the literature show that ACL reconstruction is associated with functional improvement in middle-aged patients, similar to our study. Studies are conducted using different graft types like hamstring tendon autograft, quadriceps tendon autograft, or bone–patellar tendon–bone autograft at patients older than 50 years of age have shown improved functional knee scores and high rates of return to sportive activitie.^40^, ^41^, ^42^ Corona et al.^43^ showed comparable results with younger patients at a large systematic review. Weng et al.^44^ stated patients aged older than 50 years showed similar results with patients aged younger than 30 years and showed low complication rates as well at their cohort study.

### Limitations

The short postoperative follow-up period, small sample size, and lack of comparison with control groups are among the shortcomings of our study. the fact that we could not show to what extent the concomitant cartilage lesions and meniscal pathologies affected the results is another limitation. Finally, we did not note the level at which the patients returned to sports and in which sports they were engaged.

## Conclusions

Improved functional knee scores, quality of life, and psychological status were achieved at ACL reconstruction in patients older than 50 years of age.

## Supplementary Data

ICMJE author disclosure forms
